# Formation, function, and exhaustion of notochordal cytoplasmic vacuoles within intervertebral disc: current understanding and speculation

**DOI:** 10.18632/oncotarget.18101

**Published:** 2017-05-23

**Authors:** Feng Wang, Zeng-Xin Gao, Feng Cai, Arjun Sinkemani, Zhi-Yang Xie, Rui Shi, Ji-Nan Wei, Xiao-Tao Wu

**Affiliations:** ^1^ Department of Spine Surgery, Zhongda Hospital, School of Medicine, Southeast University, Nanjing, Jiangsu Province, China; ^2^ Surgery Research Center, School of Medicine, Southeast University, Nanjing, Jiangsu Province, China; ^3^ Department of Orthopedic Surgery, The First Affiliated Hospital of Soochow University, Soochow, Jiangsu Province, China; ^4^ Department of Orthopedics, Zhongda Hospital, School of Medicine, Southeast University, Nanjing, Jiangsu Province, China

**Keywords:** intervertebral disc, notochord, notochord vacuolation, cytoplasmic vacuole, nucleus pulposus

## Abstract

Notochord nucleus pulposus cells are characteristic of containing abundant and giant cytoplasmic vacuoles. This review explores the embryonic formation, biological function, and postnatal exhaustion of notochord vacuoles, aiming to characterize the signal network transforming the vacuolated nucleus pulposus cells into the vacuole-less chondrocytic cells. Embryonically, the cytoplasmic vacuoles within vertebrate notochord originate from an evolutionarily conserved vacuolation process during neurulation, which may continue to provide mechanical and signal support in constructing a mammalian intervertebral disc. For full vacuolation, a vacuolating specification from dorsal organizer cells, synchronized convergent extension, well-structured notochord sheath, and sufficient post-Golgi trafficking in notochord cells are required. Postnatally, age-related and species-specific exhaustion of vacuolated nucleus pulposus cells could be potentiated by Fas- and Fas ligand-induced apoptosis, intolerance to mechanical stress and nutrient deficiency, vacuole-mediated proliferation check, and gradual de-vacuolation within the avascular and compression-loaded intervertebral disc. These results suggest that the notochord vacuoles are active and versatile organelles for both embryonic notochord and postnatal nucleus pulposus, and may provide novel information on intervertebral disc degeneration to guide cell-based regeneration.

## INTRODUCTION

According to systematic analysis for the Global Burden of Disease Study 2010, lower back pain (LBP) remains the leading cause of disability, affecting around 632 million people worldwide, followed by major depressive disorders [1]. It is also estimated that over 80% of the population may experience LBP at least once during their lives [2]. Intervertebral disc (IVD) degeneration (IVDD), which results in instability, stenosis, and deformity of spinal motion segments, is closely associated with a large percentage of LBP cases [3]. However, it remains unclear what initiates and propels the degenerative changes within IVD [4, 5]. Current clinical strategies, including both surgical treatments and conservative therapies, aim towards symptomatic relief rather than targeting the IVDD directly [6–8].

**Figure 1:**
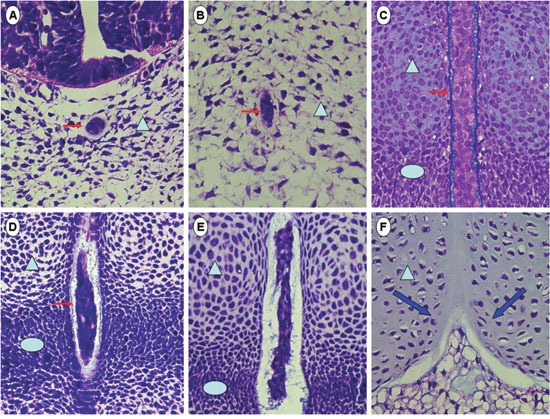
Hematoxylin and eosin staining of rat axial notochord from embryonic day 10 (E10) to E18.5 As early as E10 (**(A)**, transverse section; **(B)**, coronal section), the axial notochord (arrow) had separated from paraxial mesoderm (arrowhead), although the peri-notochord basement membrane had not fully formed. At E12 **(C)**, the axial notochord was enwrapped by a thick fibrotic sheath (arrow), lateral to which the paraxial mesoderm had patterned into non-condensed sclerotome (arrowhead) and condensed sclerotome (ellipse). From E13.5 **(D)** to E14.5 **(E)**, the notochord sheath became thinner as the non-condensed sclerotome transformed into vertebrae anlagen (arrowhead) and the condensed sclerotome into AF anlagen (ellipse), although the notochord cells had not been vacuolated at this time. The gap between axial notochord and paraxial mesoderm was widened during this stage (E13.5–14.5), which was probably caused by convergent extension and/or increased intra-notochord pressure induced by high secretion from notochord cells. From E14.5 to E18.5 **(F)**, the paired non-condensed sclerotome fused symmetrically (blue arrow) to form the vertebral body (arrowhead), whereas the notochord cells vacuolated and were squeezed into the center of IVD, probably by the pushing pressure generated from the forming vertebrae. Original magnification ×400.

**Figure 2:**
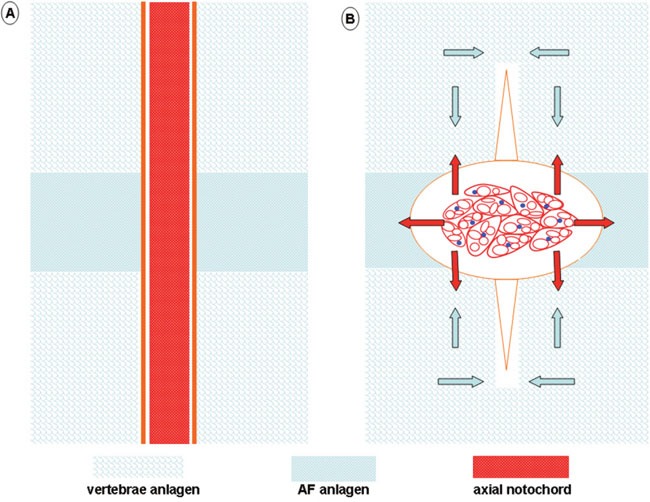
Schematic illustration of IVD formation and mechanical support from notochord vacuolation During the formation of mammalian IVD, the paired vertebrae anlagen (non-condensed sclerotome) fuses and compresses the axial notochord into the center of forming IVD, the paired AF anlagen (condensed sclerotome) expands outward and enwraps the vacuolating notochord. By generating even resistance (red arrow) to the paired inward-expanding (blue arrow) vertebral anlagen, the symmetrical morphology of vertebral body could be maintained by the vacuolating notochord. By producing a primitive swelling tension (red arrow) inside the forming IVD, the vacuolating notochord might also contribute to condense the forming AF and maintain IVD height between the growing vertebra.

A young and normal IVD is composed of three distinct components: the central gelatinous nucleus pulposus (NP), the outer fibrotic annulus fibrosus (AF), and the cartilaginous endplate (CEP) that anchors into the growth plate of the vertebral body 5, 8]. Embryonically, NP originates from the axial notochord, while AF and CEP are formed from the paraxial mesoderm [9, 10] (Figure 1, Figure 2). Studies across species have revealed that, in some vertebrates such as non-chondrodystrophic dog and rabbit, the notochordal cells persist within NP throughout most of adult life, and in these species, IVDD is significantly postponed [9–12]. By contrast, in human NP, most of the notochordal NP cells (NNPCs) have transformed into chondrocyte-like NP cells (CNPCs) before skeletal maturity, and early onset of IVDD is common in adolescents [9–13]. While a direct cause-effect relationship between the loss of NNPCs and IVDD has yet to be established, previous studies suggest that NNPCs are versatile and capable of: (1) generating offspring of CNPCs [14]; (2) attracting CEP chondrocytes into NP [15]; (3) secreting nutritional factors to rejuvenate neighboring disc cells [16, 17]; (4) stimulating chondrogenic differentiation of mesenchymal stem cells [18]; (5) inhibiting infiltration of neurons or endothelium into IVD [19, 20]; and (6) suppressing CNPC death within harsh disc niches [21]. Since the disappearance of notochordal cell resources precedes and contributes to degenerative changes within IVD [9–12], it is possible that the early process of IVDD, at least the disturbance of homeostasis in NP, is initiated by the loss of NNPCs and could be targeted by preserving them.

**Figure 3:**
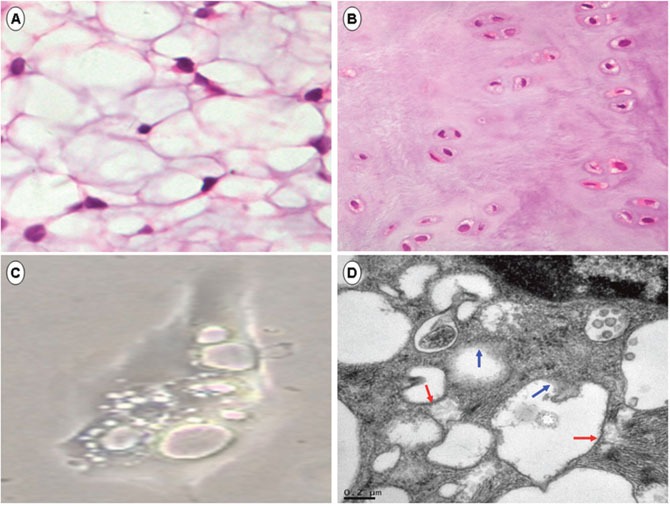
Exhaustion of notochord nucleus pulposus cells Hematoxylin and eosin staining of neonatal **(A)** and aged **(B)** rat NP showed the replacement of vacuolated notochord NP cells (NNPCs) by non-vacuolated chondrocytic NP cells (CNPCs) and fibrocartilaginous extracellular matrix during natural aging. In monolayer cultures **(C)**, the NNPCs were rich in giant cytoplasmic vacuoles. On transmission electron micrographs **(D)**, the vacuole membrane (red arrow) in NNPCs isolated from adult rat was partially or completely lost (blue arrow), suggesting that a de-vacuolation process may also contribute to the loss of NNPCs. Original magnification ×400 for **(A–C).**

Compared with other musculoskeletal cells, NNPCs are unique in exhibiting large and abundant cytoplasmic vacuoles [11, 22] (Figure 3). Transmission electron micrographs revealed that some of the large vacuoles could be 20 μm in diameter and account for 80% of total cell volume, presumably containing electron-lucent and hyposmotic solution [22, 23]. It is now known that these vacuous inclusions are membrane-bound, express ion pumps, and are generated by a vacuolation process early in embryogenesis [22–25]. Interestingly, within NNPCs isolated from adult rat IVD, we noticed that the vacuole membrane could be partially or completely lost (Figure 3D), suggesting that decreased synthesis and/or an increased degradation of endomembrane may cause de-vacuolation and contribute to the exhaustion of cytoplasmic vacuoles. In other words, these cytoplasmic vacuoles may be generated for a specific reason and maintained by specific signals. Dysregulation of these signals initiates or accelerates the loss of NNPCs.

This review focuses on our understanding of the formation, function, and exhaustion of notochordal vacuoles, aiming at characterization of the vacuolation-related mechanism and pathways. Being the central regulator of embryonic development, axial notochord also releases various signaling molecules to orchestrate the construction of both the vertebral column and the paraspinal organs [10, 26]; more detailed information on notochord-derived signals and their roles in specifying the central nervous system, left-right asymmetry, and arterial-venous identity can be found in several recent reviews [25, 27, 28].

### The evolutionary road leading notochord to IVD

The discovery of *Yunnanozoon* in Early Cambrian Chengjiang fauna (Maotian Hill, Yunnan, China) revealed that chordates, which share a defining characteristic of a rod-like notochord, have lived on earth for more than 525 million years [29]. In present-day mammals, including humans, the notochord structure remains evident during embryogenesis and forms the NP of postnatal IVD [9, 10] (Figure 1). However, to become part of the vertebral column, the axial notochord has experienced millions of years of evolution and changed in both morphology and functionality [26, 30]. Recent findings in marine annelids indicate that the most ancestral notochord may be an axial muscular structure, termed the axochord, and is positioned between the central nervous system and axial blood vessel in the protostome-like ancestors [31, 32]. Possibly by losing the muscle traits and gaining a vacuolating property, the axochord may have evolved to become the rod-like notochord in modern vertebrates [31, 33]. In support of this hypothesis, the notochord cells of cephalochordate (lower chordate) maintain part of their muscular traits by containing transverse myofilaments [34, 35]. Besides, while the muscular phenotype may have regressed within the notochord of tunicates (sister group of vertebrates), the intracellular vacuolating process of vertebrates has yet to be fully developed by the tunicate notochord, in which extracellular vacuoles are generated and coalesced to form a running lumen, while the non-muscular notochord cells assume an endothelial-like shape and surround the notochord [36].

For invertebrate chordates (cephalochordate, tunicate), the notochord mainly functions as a hydrostatic skeleton to allow undulating swimming, which persists throughout life in cephalochordates but exists only in the larvae of tunicates [34–36]. For early vertebrates such as lamprey and sturgeon, the notochord also persists throughout life, but becomes the attachment site for the primitive vertebral column [30, 37] (mainly the vertebral arches, probably because they evolved earlier than vertebral bodies). Studies on the evolutionary origin of the vertebral body have revealed that, in many fish species (elasmobranchs, teleosts), the notochord generates primitive cartilaginous vertebrae, namely, the chordacentra, which together with the subsequent sclerotome-derived perichordal centra form the vertebral body [30, 38]. A typical example comes from the zebrafish in teleosts, which utilizes notochord rather than somites to secrete bone matrix and build vertebra [39]. Interestingly, as evolution progressed, although most vertebrates chose to generate vertebrae exclusively from the paraxial mesoderm, the axial notochord is not necessarily destined to become NP within most non-mammalian vertebrates [40, 41], such as avian and amphibians, suggesting that a well-structured IVD consisting of both AF and NP may form only in highly evolved mammalian organisms.

### Vacuolation of notochord cells

#### When do notochord cells vacuolate?

Embryonically, notochord arises from a specific cell mass within the dorsal organizer, which in a notochord-independent manner generates the cranial-caudal axes and defines the dorsal-ventral identities in embryos [26, 28]. Prior to vacuolation, the mixed organizer cells first require a chordamesoderm specification under coordinated nodal signaling, stimulating the superficial layers of the dorsal organizer into notochord induction [42]. Later, during gastrulation and neurulation, the notochord grows longer, narrower, and thicker after the following coordinated steps [26, 43, 44]: (1) during late gastrulation, notochord cells become distinct from somitic mesoderm and the two are demarcated by a emerging boundary that accumulates laminin, fibronectin, fibrillin, and collagen; (2) from the end of gastrulation to early neurulation, as mediolateral intercalation and convergent extension proceed, the notochord becomes narrower and taller, changing into the trapezoidal shape on transverse sections; (3) from middle to late neurulation, notochord cells enlarge evenly and the trapezoidal notochord transforms into a cylindrical structure surrounded by a thick notochordal sheath, following which vacuolation initiates within the cranial notochord and progresses caudally. In addition to the convergence and extension, notochord can also be lengthened by recruiting new chordamesoderm cells into its caudal end [44]. These observations suggest that vacuolation is the final step in notochord differentiation and may rely on the preceding chordamesoderm specification, convergent extension, formation of enwrapping notochord sheath, and lastly the spatiotemporal activation of vacuolating signals within the axial notochord.

### Notochord vacuoles are generated by post-Golgi trafficking pathways

Generally, cytoplasmic vesicles are supplied by post-Golgi biosynthetic trafficking and endocytic trafficking of the internalized membrane [45, 46]. Briefly, along the post-Golgi trafficking pathways, secretory cargo is synthesized within the endoplasmic reticulum (ER) and transported into the Golgi complex by coat protein (COP)-II coated vesicles, after which the cargo is sorted and delivered by clathrin-coated vesicles into early endosomes, late endosomes, and finally lysosomes, or into secretory granules and plasma membrane for release. As shown in adult rat notochordal cells, the cytoplasmic vacuoles may require a continuous supply of endomembrane to maintain their vacuolated morphology (Figure 3D). In zebrafish, it has been shown that the post-Golgi trafficking rather than endocytosis contributes to the biogenesis and maintenance of notochord vacuoles [47]. Specifically, the zebrafish notochord vacuoles are lysosome-related organelles that would fail to vacuolate if ER to Golgi transport is blocked by brefeldin [47] or a COP-I mutation [48]; for sufficient vacuolation, Rab32a-mediated late endosomal trafficking [47], H^+^-ATPase-dependent acidification [47], and Scarb2-mediated lysosomal maintenance are also required [49].

Nevertheless, the content of notochordal vacuoles and the sorting mechanism through which cargo is routed into vacuoles rather than lysosomes remain unclear. Findings from canine [22, 23], zebrafish [47], and rat notochord cells (Figure 3) showed that the cytoplasmic vacuoles are electron-lucent and negatively stained by eosin, Periodic Acid-Schiff, Oil Red O, and Alcian Blue, suggesting that the content could mainly be water. Based on the expression of both sodium/potassium ATPase and H^+^-ATPase on the vacuole membrane [23, 47], there may be dynamic across-membrane exchange of H^+^ and alkali ions that drives an inflow of water to inflate these cytoplasmic vacuoles. However, while the aquaporins that mediate water transport have been detected on the cell membrane of rat NP cells, it remains unknown whether they are also localized on the vacuolar membrane and function in the transport of water [50]. One *in vitro* study of canine NNPCs suggested that the vacuoles contain hypo-osmotic solution that could be released to dilute cytoplasm in cases of hypotonic stress [23]. Since the glycosaminoglycan side chains of proteoglycans are hydrophilic and promote water retention [4, 51], the extracellular space of NP is likely hyper-osmotic and there may be osmotic gradients across the cellular and vacuolar membranes. Although it remains unclear how NNPCs respond to and tolerate osmotic gradients *in vivo*, the contacting clusters of notochord cells and their densely packed cytoplasmic filaments [22] may help strengthen the membrane and withstand hydrostatic pressure generated by the inflating vacuoles.

### Notochord sheath is a prerequisite for vacuolation

Following chordamesoderm specification in the superficial layers of dorsal organizer, the notochord precursors will initiate the development into two morphologically distinguishable cell types: the inner vacuolating cells and the outer non-vacuolating epithelium-like cells, lateral to which a peri-notochord acellular basement membrane (PNBM) or notochord sheath (Figure 1C) is forming [26, 42]. Notch signaling is known to be activated by Mind bomb (Mib)-Jag1, which functions in cell-fate switching from vacuolating to non-vacuolating cells, determining the number of cells to be vacuolated and the thickness of the notochord sheath surrounding them [52].

Ultrastructurally, PNBM is composed of three different laminar layers: the inner laminin-rich layer, and the intermediate and outer collagen-rich layers with their fibers oriented orthogonal to each other [48]. Owing to the need for a well-structured PNBM to withstand the increasing intra-notochord pressure (Figure 1D, 1E), vacuolation will be disturbed once the components and/or structures of the notochord sheath are disrupted. As shown in zebrafish with mutations in the *grumpy* and *sleepy* genes, which encode the β1 and γ1 chains of laminin-1, respectively, the inner layer was missing while the entire PNBM was disorganized and finally lost, enwrapping notochord cells that were significantly less vacuolated [53]. Similar morphogenesis was also observed in zebrafish deficient in the α1α4 or α1α5 chain of laminin [54], suggesting that the latter may be essential for constructing PNBM and potentiating vacuolation. Recent findings in *Xenopus laevis* supported this hypothesis by showing that the laminin within PNBM bonded to the dystroglycan and myosin IIA expressed by notochord cells, forming a laminin-dystroglycan-myosin IIA complex to maintain cytoskeleton integrity during vacuolation [43]. Besides, by generating phosphotyrosine signals, the binding of laminin to dystroglycan may also contribute to activate notochord vacuolation [43], presumably through cross-talking with the post-Golgi trafficking pathways [47].

In addition to laminin, PNBM is also composed of fibrillin, fibronectin, proteoglycan, and various collagens [43]. Of these, (1) depletion of type XV collagen (Col-15) ruptured the inner layer of PNBM and disorganized its fibrous middle and outer layers [55, 56]; (2) mutation of fibrillin-2 resulted in a notochord sheath with a narrow outer layer but normal inner and middle layers [57, 58], whereas loss of Emilin-3 [59] (elastin microfibril interface-located protein-3) or the α1 chain of Col-8 [60] disrupted only the laminin-rich inner layer; and (3) knockdown of the α1 chain of Col-27 [61] or the copper-dependent lysyl oxidases that catalyze the crosslinking of elastin and collagens induced no significant change in the three layers of zebrafish PNBM [62]. Consistent with the severity of disorganization within the notochord sheath, failure of vacuolation occurred only in the zebrafish with depletion of Col-15 [56], although a kinked notochord was detected in all the embryonic mutants [57, 59–62]. Taken together with laminin [53, 54], a well-structured notochord sheath may not only provide architecture and signaling support for vacuolation, but also generate appropriate strength and rigidity to withstand the vacuole-derived pressure, contributing to straightening during elongation of the growing notochord.

Being a demarcating boundary that segregates axial notochord and somitic mesoderm, the histocytological origin of the three layers of PNBM has long been debated [25, 52]. Early graft assays of the dorsal organizer between laminin-1 mutants and wild-type zebrafish showed that the transplanted organizer could generate a new notochord surrounded by laminin-1 in either wild-type or mutant recipient [53], suggesting that both the axial notochord and the paraxial mesoderm contribute to the laminin-rich inner layer. A similar dorsal organizer exchange between wild-type and COP-I mutant zebrafish embryos, in which the outer two layers were absent from PNBM, showed that the mutant organizer yielded only disorganized PNBM, while the wild type formed a normal notochord sheath [48]. Thus, the outer layers of PNBM were constructed mainly by the axial notochord. An ultrastructural comparison between the inner vacuolating and outer non-vacuolating notochord cells demonstrated that the latter contained abundant rough ER, and once they were specified into vacuolation, the middle layer of PNBM would be narrowed [52], suggesting that the outer epithelium-like notochord cells may be responsible for constructing the collagen-rich middle layer of the notochord sheath. However, it remains unclear whether and how the prospective vacuolating notochord cells contribute to the inner and outer layers of PNBM. Notably, in zebrafish with blockage of ER to Golgi transport, the laminin-rich inner layer could still be formed [48], suggesting that there may be a vesicle-trafficking-independent mechanism to guarantee laminin biosynthesis. In addition, the inner layer of PNBM may have been properly constructed before the high secretory demand in the inner vacuolating cells.

### Convergent extension potentially affects vacuolation

Convergent extension is a highly conserved mechanism that shapes notochord, somite, and spinal cord during early embryogenesis [44, 63, 64]. Studies across species have revealed that convergent extension is driven by polarized cell movements that pattern cell intercalation and can proceed independent of adjacent tissues [64]. To date, the planar cell polarity pathway, which originally controls the polarity of single hairs on each epidermal cell of the fly wing, has been identified as the signal basis that also underlies the planar mesenchymal cell polarity in a variety of chordates [63, 64]. Moreover, for the proper polarization of cell movements within a developing notochord, accumulation of fibrillin at the presumptive notochordal-somitic boundary [58, 65], cell-cell interaction mediated by integrin(α5β1)-fibronectin binding [66], and rearrangement of actin cytoskeleton induced by β-dystroglycan [43] are also required. Since most of these matrix components will be integrated into the prospective PNBM [43], failure of convergent extension may hinder notochord vacuolation by impairing the formation of a well-structured notochord sheath. In support of this, when mediolateral intercalation was disturbed by mutating the juxtamembrane and internal sites of β-dystroglycan, an enlarged but shortened notochord with smaller vacuoles was formed in *Xenopus* embryos [43]. In fact, probably because convergent extension progresses coordinately in both axial notochord and paraxial structures [63, 64], failure often leads to severe defects in overall embryonic morphogenesis, including but not limited to non-fusion of the neural fold [67], disorganization of heart and blood vessels [68], and even complete loss of somite and notochord during early gastrulation [68, 69]. Therefore, further studies are required on the notochord sheath and vacuoles in deformed embryos to identify the cause-effect relationship between convergent extension and notochord vacuolation.

### Functionality of notochord vacuolation

In addition to the mechanical supports that elongate and straighten the cranial-caudal axes of developing embryos, the notochord secretes a variety of inductive factors to pattern the development of adjacent neural tube, myotome, and sclerotome, which will eventually enwrap and engulf the signaling notochord [10, 25–27]. Notably, once the notochord sheath has been formed and notochord cells are vacuolated, release of these notochord-derived signal factors such as Col-2 and Hedgehog family proteins will be reduced [25, 26]. As shown in developing rat embryos (Figure 1C–1E), both spinal cord and paraxial sclerotome were properly patterned before the notochord vacuolated, suggesting that cytoplasmic vacuoles may commit to terminate rather than stimulate the patterning effects on peri-notochord tissues. Evolutionarily, the mechanical role of notochord will persist in adults as the hydroskeleton for invertebrate cephalochordate and some early vertebrates [26, 30] (lampreys, sturgeons, paddlefish); however, for postnatal mammals, it remains unclear whether the vacuolating notochord will continue to provide mechanical and/or signal support during and after the vacuolated cells are compartmentalized into the center of IVD.

### Mechanical support

During IVD formation, the condensed portion of the paraxial sclerotome expanded laterally to develop into AF, while the non-condensed sclerotome grew medially and fused to form the vertebral body, by which the axial notochord was compartmentalized into the center of the prospective disc and became NP [9, 10] (Figure 1, Figure 2). Two illustrative models, namely, the “pressure” model and the “repulsion/attraction” model, are available to depict the transition of notochord into NP [70]. Briefly, the “pressure” model shows that notochord cells are passively squeezed into IVD by the pushing force derived from the forming vertebrae, whereas in the “repulsion/attraction” model, the boundaries between different compartments of IVD may have been specified beforehand by the spatiotemporal expression of sorting and positioning signals. Although more studies are required to validate these models, the kinked spine of vacuolation-deficient zebrafish suggested that the pushing force of invading prevertebral cells should be properly opposed by the vacuole-derived tensions to maintain symmetrical morphology in the developing trunk [47]. This was also supported from the dorsally bent spine of *scarb2a* insertional mutants [49], in which the ventral side of notochord sheath was anomalously thickened, possibly disturbing the mechanical equilibrium along the ventral-dorsal axis.

Considering that constructing the mammalian vertebral column is much more complicated and orchestrated than building fish spine [30, 40], it is possible that the mechanically supportive role of the notochord is evolutionarily conserved among chordates and contributes to the construction of a disc-connected spine in the following ways (Figure 2): (1) By generating even resistance to the paired inward-expanding vertebral anlagen, the symmetrical morphology of the vertebral body is maintained while the vacuolating cells themselves are propelled into prospective IVD. (2) By producing a primitive swelling force inside IVD, the condensed phenotype of the AF anlagen is preserved while the height of IVD is maintained between the growing cranial and caudal vertebrae. (3) Prior to a sufficient accumulation of water-binding matrix within NP, the hydrated vacuoles function to provide tissue rigidity and absorb mechanical loading. For validation, unilateral resection of non-condensed sclerotome may confirm whether the notochord is compressed or attracted into NP; by quantifying the cytoplasmic vacuoles within different segments of the embryonic spine, the relationship between disc height and notochord vacuolation could be understood. In rat embryos, the generation of IVD was accompanied by thinning and vanishing of the notochord sheath (Figure 1D–1F). Although further validations in other mammals are required, the disassembly of thick PNBM could suggest that the notochord-derived tension counteracts the pushing force of vertebrae during IVD formation.

### Signaling function

In neonatal rat NP, vacuolated NNPCs are the primary if not exclusive cell resources, which accumulate much less matrix than adult NP populated with both NNPCs and non-vacuolated CNPCs [5]. As aging progresses, the CNPCs increase to replace NNPCs, while more cartilaginous matrix is produced within NP (Figure 3A, 3B). *In vitro* cell live imaging has shown that CNPCs are generated by NNPCs and proliferate much faster than the vacuolated cells [14], suggesting that cytoplasmic vacuoles within NNPCs crosstalk with some signals to check cell proliferation. Being a defining characteristic that morphologically distinguishes NNPCs from CNPCs, the giant vacuoles not only occupy most of the cell volume, but also promote contact between cells and form large clusters [22], probably initiating contact-induced cell quiescence mediated by Hippo-Yap (Yes-associated protein) pathways [71–74]. Briefly, at low cell densities, the core components of Hippo signals (protein kinases Mst1/2 and Lats 1/2) are weakened to allow nuclear accumulation of Yap, which promotes cell proliferation by co-activating the transcription factor Tead1-4, whereas high cell densities induce strong Hippo signaling that suppresses cell growth by promoting cytoplasmic retention and ubiquitination-mediated degradation of Yap [71–74]. In contacting cells, the high density is sensed and transduced to Hippo by cell-cell junctional proteins such as angiomotin protein complex and α-catenin [75]. Studies on the three-dimensional architecture of canine NNPCs showed that these vacuolated cells were interconnected via gap junctions and resisted mechanical disruptions [22]. In addition, they likely maintained elevated and tightened cell-cell gap junctions in the crowded cytoplasmic vacuoles, which could activate Hippo-Yap pathways to check NNPC proliferation. Another link between vacuoles and Yap could be through the remodeling of cytoskeletal proteins, such as actin and microtubules, which shape cell morphology and regulate protein kinases of Hippo signals [73, 74]; however, although the cytoskeleton within NNPCs is significantly compressed and distorted by cytoplasmic vacuoles, how this modulates the activation of Yap and controls the growth kinetics of vacuolated cells remains unclear.

### Exhaustion of notochord vacuoles

Unlike eruptive vacuolation within a short period during neurulation [44], vacuolated NNPCs experience age-related and species-specific exhaustion postnatally [41]. Aside from the restrained proliferative capacity that may compromise the supplementation of new vacuolating cells [14], several other factors that contribute to reduce the viability of NNPCs have been identified, as follows: (1) NNPCs are less resistant to mechanical stress [76, 77]. (2) Compared with CNPCs, NNPCs are more metabolically susceptible to nutritional deficiency [78]. (3) NNPCs constitutively express both Fas and Fas ligand (FasL), which could induce apoptosis through autocrine or paracrine interactions [76]. Moreover, in rat lumbar IVD, we noticed that both the number and the diameter of notochord vacuoles decreased as aging progressed [8], suggesting that a chronic process of de-vacuolation may underlie the age-related exhaustion of vacuolated cell resources.

At this time, although notochord vacuoles have been shown to be lysosome-related organelles originating from post-Golgi trafficking [47], what potentiates de-vacuolation and how these cytoplasmic vacuoles are exhausted within IVD remain unclear. Cues to this enigma are expected to be provided by comparative studies between early vertebrates that preserve the notochord throughout life and mammals in which notochord cells vacuolate to build IVD, but de-vacuolate once the vertebral column (the updated version of axial skeleton) takes over mechanical loading and absorbing.

First, it is possible that the vacuole-derived tension can no longer afford the increasing compression loaded onto postnatal IVD, which breaches the vacuole membrane and encourages the transition of NNPCs into CNPCs to increase the accumulation of cartilaginous matrix within NP. In support of this hypothesis, dynamic hydrostatic pressurization has been shown to accelerate de-vacuolation and increase proteoglycan-rich matrix in cultured porcine NP [80]. Accordingly, the accumulative effects of mechanical loading may account for exhaustion of notochord vacuoles during aging, whereas the bipedalism of humans could induce excessive compression and lead to the premature loss of NNPCs [41, 81–83]. Since intradiscal pressure is associated with the size and shape of IVD, the stability and force of muscle or ligament, as well as the integrity and functionality of zygapophyseal joints between vertebrae [84], it would not be surprising to find that NNPCs and their cytoplasmic vacuoles are exhausted inhomogeneously by diverse mechanical stresses among different animals and/or at different segments.

Another possibility concerns the avascular nature of IVD [5, 85], which likely fails to provide sufficient nutrients or energy to maintain high levels of post-Golgi trafficking and vesicle biogenesis. In addition, in mouse notochord with conditional deletion of hypoxia-inducible factor-1α (HIF-1α), normal vacuolation occurred during IVD formation, but then de-vacuolation and disappearance occurred rapidly thereafter [86]. This suggests that once vacuolating cells are enwrapped by IVD, the nutrition and oxygen supplied to them may be significantly reduced, after which HIF-1α-dependent metabolic adaptation must be required to maintain or decelerate the loss of cytoplasmic vacuoles. On the other hand, within the avascular IVD, self-protective autophagy, which relies on the isolation of membrane and lysosome to recycle dysfunctional proteins [87, 88], can be activated to provide additional metabolic substrates for cellular adaptations [89]; since the notochord vacuoles are lysosome-related organelles [47], it is possible that starvation-induced autophagy competes to inhibit the vacuolating pathways. Other studies have shown that notochord vacuoles could be abated by hypotonic stresses [23] or the weakening of canonical Wnt signals [90]. Given that matrix components determine tissue osmolality [91–93] while oxygen tension regulates β-catenin expression [94], it is possible that these factors function downstream of the increased mechanical loading and the avascular microenvironment to de-vacuolate NNPCs. In addition, as the notochord cells in normoxic cultures (without hypoxia and compressive loading) also lose vacuoles [14], there may be other unidentified factors exhausting the cytoplasmic vacuoles of NNPCs.

### Conclusions and outlook

**Figure 4:**
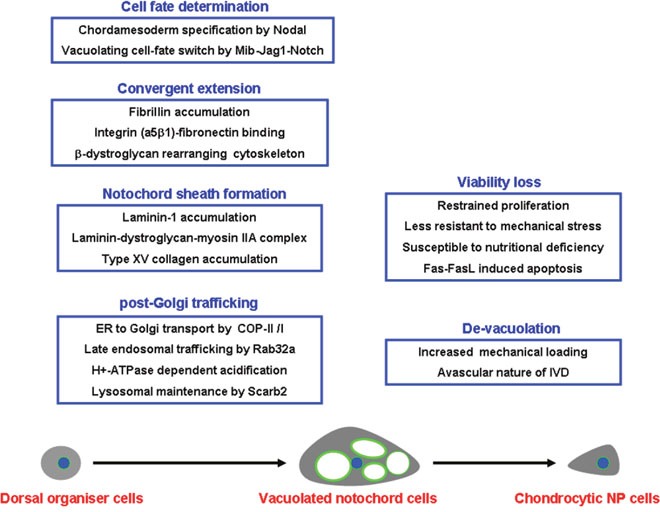
Summary of the signal basis underlying the generation and exhaustion of notochord vacuoles Notochord vacuolation is a highly coordinated and orchestrated process that requires vacuolating specification from dorsal organizer cells, intact convergent extension, formation of notochord sheath, as well as sufficient post-Golgi trafficking within the notochord cells themselves. After squeezing into the IVD, the vacuolated notochord NP cells can be exhausted by inferior cell viability and a chronic process of de-vacuolation, which may result from the increased mechanical stress and avascular nature of postnatal IVD.

Notochord vacuolation is evolutionarily conserved in vertebrate embryogenesis and is orchestrated by a series of vacuolating signals (Figure 4). Vacuolated notochord cells are the major source of mechanical support that is not only required for the axial elongation of growing embryos, but also for morphogenesis of the vertebral column, although the latter is diversified among different species regarding morphology and methods of disposal of the axial notochord. In mammalian spine, NNPCs are likely squeezed into the center of IVD, where the avascular nature and mechanical stress may potentiate the exhaustion of vacuoles and promote the transformation into CNPCs. By unfolding the signaling pathways that mediate the formation and function of notochord vacuoles, the age-related exhaustion of NNPCs and particularly the premature de-vacuolation and degeneration in human IVD could be better understood.

Since notochord vacuoles are active and versatile organelles for both embryonic notochord cells and postnatal NNPCs, targeting de-vacuolation or preserving vacuolating signals is expected to provide novel clues to decelerate IVD degeneration. Nevertheless, it should be noted that our current understanding of notochord vacuoles is mainly based on observations in vertebrates without typical IVD (mostly zebrafish), and caution should be taken when translating these results into the pathophysiology of IVD in mammals (especially humans); future studies on the embryonic formation of the mammalian vertebral column are required to validate the signaling network and biological function of notochord vacuolation. Besides, the evolutionary map of the notochord [33] and the early exhaustion of human NNPCs [84] suggest that an IVD composed of central gelatinous NP and outer fibrotic AF is not customized specifically for humans who walk and work upright. As our IVD may inherently experience more mechanical stress (the intradical pressure of human L4/5 disc was 0.1 Mpa in prone position but increased to 0.5 Mpa when standing and 2.3 Mpa when lifting a weigh [81]), it should be re-evaluated whether cell-based disc repair (e.g. cell transplantation [8], gene therapy [95, 96]) is better than mechanical reconstruction strategies (e.g. IVD arthroplasty [97], disectomy [6, 7], interspinous fixation [98], and pedicle-based dynamic stabilization [99]) in treating a degenerated IVD, especially when a long-term positive outcome is desired.
